# “Balance T” device improves balance confidence and performance in repeated measures study

**DOI:** 10.1016/j.tjfa.2025.100113

**Published:** 2025-11-27

**Authors:** Courtney Walker, June Nicholas, Crystal Szczesny, Jeremy Walston, Yuri Agrawal, Michael C Schubert

**Affiliations:** a Department of Otolaryngology— Head and Neck Surgery, Johns Hopkins University School of Medicine, Baltimore, MD, USA; b Hopkins ElderPlus, Johns Hopkins University School of Medicine, Baltimore, MD, USA; c Division of Geriatrics Medicine and Gerontology, Johns Hopkins University, Baltimore, MD, USA; d Johns Hopkins Asthma and Allergy Center, Baltimore, MD, USA; e Department of Otolaryngology— Head and Neck Surgery, University of Colorado Anschutz Medical Campus, Aurora, CO, USA; f Department of Otolaryngology— Head and Neck Surgery, Laboratory of Vestibular NeuroAdaptation, Johns Hopkins University, Baltimore, MD, USA; g Department of Physical Medicine and Rehabilitation, Johns Hopkins University, Baltimore, MD, USA

**Keywords:** Balance, Exercise, Older adults, Geriatrics, Frailty

## Abstract

**Purpose:**

The purpose of this research is to determine if balance can be improved in older adults from exercising at home using a novel piece of exercise equipment called the “Balance T”.

**Method:**

Fifty older adults (77.9 ± 6.7 years old) participated in a four-week home exercise, open-label clinical pilot study investigating ability to improve balance using the Balance T device. Pre- and post-intervention assessments included the Activities-specific Balance Confidence (ABC) Scale, Short Physical Performance Battery (SPPB), Timed Up and Go (TUG), Clinical Test of the Sensory Interaction on Balance (CTSIB), Balance T Change Score (BTCS), and a user-opinion survey. Statistical analyses comprised parametric and non-parametric tests for pre-post exercise comparisons as well as evaluated balance differences across frailty status using established frailty-defining criteria.

**Results:**

Frail participants at-risk for falls exhibited greater improvement in balance confidence compared to robust participants, with a mean change of 5.28 ± 12.9 (*p* < 0.001). In addition, each performance variable of balance showed clinically significant and meaningful changes after completing the Balance T exercise program. Participants reported the Balance T device as easy and safe to use with benefits gained at improving their balance.

**Conclusion:**

The Balance T device proved feasible for home use, showing preliminary evidence of improved balance performance and confidence, particularly among frail adults.

## Highlights

• **Knowledge gap/clinical motivation:** Impaired balance in older adults is a major contributor to falls, with limited accessible at-home intervention available to improve balance and reduce fall risks.
• **Key findings:** The Balance T device significantly improved balance confidence and performance in older adults, particularly those at risk for falls, with participants reporting it as easy and safe to use.
• **Clinical relevance/application:** The Balance T offers a home-based solution for improving balance confidence and performance, especially for frail older adults, to reduce fall risks and improve life quality.

## 1 Introduction

Impaired balance is a leading cause of falls among older adults, with significant implications for their health and quality of life. In the United States, one in four older adults experiences a fall each year, and in 2018 alone, there were 36 million reported falls [1]. Unintended falls are the most prevalent type of accidental injury and the most common cause for accidental fatalities in the United States [2]. The consequence of falling on older adults are substantial, as evidenced by the fact that 3 million individuals are treated annually in emergency departments for fall-related injuries, and 20 % of these falls are severe, including head trauma or hip fracture [3,4]. The aging population in the United States is growing at an unprecedented rate, and this demographic shift has raised public health concern for fall-related risks and their associated health implications like mortality, morbidity, and high costs [5].

As we age, our bodies experience transformations that compromises physical resilience. Muscle mass and bone density diminish and manifest as altered posture, unstable gait, and reduced static balance – all of which elevates the potential for fall-related incidents among older adults [5]. To improve functional capacity and prevent falls in older adults, the WHO guidelines on Physical Activity and Sedentary Behaviour recommends physical activity with an emphasis on functional balance and strength training [6]. However, balance confidence, the self-perceived ability to maintain balance and avoid a fall, worsens with age and contributes to activity restriction and a decline in physical function in older adults [7]. While assistive devices (ADs) may be a good alternative to provide confidence in safe participation of physical activity, they are not commonly prescribed unless individuals are seen clinically for a mobility impairment. Furthermore, the effect of using an AD in healthy older adults for the purpose of improving balance is unknown.

In response to this critical issue, researchers at the Johns Hopkins School of Medicine designed “The Balance T,” a mechanical exercise device aimed at improving balance and reducing fall risks in older adults by targeting and improving balance confidence. Balance confidence is a significant and modifiable predictor of falls. Lower balance confidence contributes to fall avoidance behaviors, including activity restriction [7]. The purpose of this study was to investigate how effective the Balance T Device is for improving both balance confidence and function in older healthy and frail adults.

## 2 Methods

### 2.1 Participants

Fifty adults aged 55 – 100 years and able to ambulate were recruited from the Johns Hopkins Artificial Intelligence and Technology Collaboratory for Aging Research at The Johns Hopkins University School of Medicine. Each was categorized for frailty status based on Fried et al.’s frailty-defining criteria used to assess fall risks in older adults [9]. After confirming their understanding of the research study, including purpose and assessment, participants provided written informed consent approved by the Johns Hopkins Medicine Institutional Review Board (IRB00364181). Participants were initially seen in person at the Healthy Aging Studies Unit (HASU) on the Johns Hopkins Bayview Medical Campus where they received the Balance T exercise device, orientation and training in its use, illustrated exercise brochure including weekly progressions to be completed at home for four weeks, instructions on self-assessment of balance, and opportunity to provide feedback. The Balance T device is customizable to participant height and difficulty in training.

This study is an A-B study using phase change without reversal where the two a priori phase sequences for all participants were baseline and Balance T exercise intervention. Balance was evaluated in the clinic before and after completing the four-week at-home exercise program. In addition, participants were instructed in how to measure their own balance improvement at home. During the course of the home exercise program, participants engaged with the study’s Board-Certified Clinical Specialist in Neurologic Physical Therapy (JN) through weekly phone calls to review prescribed exercises, assess compliance, and address any concerns. Inclusion criteria were 50–90 years old and status as an independent community dweller. Exclusion criteria included acute medical or neurologic illness, limited mobility, use of a wheelchair, and inability to participate in the study procedures.

Clinical outcomes were assessed in the HASU by Senior Research Program Coordinator, (CS), whom conducted a comprehensive battery of validated measures to evaluate the various dimensions of balance and functional mobility. The four balance outcomes include the Activities-specific Balance Confidence (ABC, subjective measure of balance confidence) Scale, Short Physical Performance Battery (SPPB, assess lower extremity function in standing and gait tasks), Timed Up and Go (TUG, time to walk 10 m and turn 180d before returning to sit), and the Clinical Test of the Sensory Interaction on Balance (CTSIB, stand eyes open/closed on firm/foam surface). These four measures were conducted in clinic both pre- and post-completion of the Balance T home exercise program. The research team developed a novel measure of the change in balance using the data collected at home by the participants, called the Balance T Change Score (BTCS). The BTCS includes nine metrics capturing lower body strength, static, and dynamic balance capacity (Table 1). The BTCS yields a composite score between 0 and 9 where higher scores indicate greater change and scores below 5 indicate less change. An increase in time for tasks that involve standing balance, such as standing with eyes open or closed on a firm surface, and standing on one leg with eyes open or closed indicate improved balance and are awarded a score of 1 for each metric where increased duration is observed. Conversely, for tasks that assess dynamic balance and mobility, such as the Five Times Sit-to-Stand Test, Timed Up and Go Test, and Timed Up and Go Test with Step Over, improved performance is indicated by a decrease in time. These tasks are scored with a 1 for each metric where time is reduced, as shorter duration reflects improved balance. The total possible score for the BTCS is 9, representing the maximum possible improvement across all assessed areas of balance and lower body strength.

**Table 1:** Nine items of the static and dynamic balance outcomes collected at home as part of the self-assessed Balance T change score (BTCS).

Test Item	Ideal Score/Unit of Measure	Improvement
Eyes Open Firm	Time for 30s	Greater
Eyes Closed Firm	Time for 30s	Greater
SOLEO Right	Time for 30s	Greater
SOLEO Left	Time for 30s	Greater
SOLEC Right	Time for 30s	Greater
SOLEC Left	Time for 30s	Greater
FTST	Total time	Lesser
TUG	Total time	Lesser
TUG with step-over	Total time	Lesser

Abbreviations: SOLEO, Standing on Leg Eyes Open; SOLEC, Standing on Leg Eyes Closed; FTST, Five Times Sit-to-Stand Test; TUG, Timed Up and Go Test.

Finally, we created a User Survey to gather qualitative and quantitative feedback on usability, satisfaction, and concerns/challenges from participants regarding their experiences with the Balance T device. The survey was delivered in a physical, paper-based format prompting numerical ratings as well as open-ended responses on device usage experience, usability, performance, satisfaction, and overall impressions (**survey available as supplemental data**).

### 2.2 Balance T exercise regimen

**Fig. 1:**
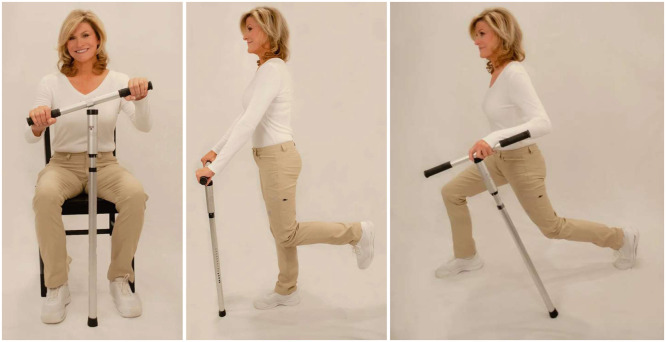
Example of the balance T exercise device being used in sit, stand, and walking.

The device involves a simple design as a T-bar joined by an articulating joint that mates a single vertical limb with a rubber foot attached to a horizontal handlebar that freely rotates 360 degrees and can be adjusted in roll tilt (Fig. 1). The Balance T home exercise program involves both static and dynamic balance exercise by use of the device’s mobile joints that place the user in a state of controlled instability. For more detail of the home program, please see **Supplemental data**.

### 2.3 Data analysis

The CTSIB and ABC outcome measures were not normally distributed, thus Wilcoxon signed rank test and Mann Whitney U test were used to compare pre to post-test values. Otherwise, all other outcome measures used parametric analysis (ANOVA or T-Test, Microsoft Office LTSC Professional Plus 2021). Alpha levels were set at *p* < 0.05. To further analyze whether the Balance T exercise program had differing effects on the cohort’s balance outcomes on those participants with a heightened fall risk, frailty-defining criteria classified participants in two frailty status levels; robustness (*n* = 14) and having some level of frailty that labeled them “at-risk” of falls (*n* = 33) [9].

A formal power analysis was not conducted for two reasons: 1. The study was designed as an exploratory, hypothesis-generating investigation rather than a definitive efficacy trial and 2. A sample size of 50 participants was selected to provide adequate precision for estimating effect sizes and feasibility metrics to inform a future, fully powered study.

## 3 Results

Of the 50 enrolled participants, three withdrew from the research study within a week of enrolling due to medical reasons. Thus, 47 individuals completed the baseline and Balance T exercise regimen (men: *n* = 13, age: 77.9 ± 6.7 years; Table 2). To examine the unique effects of frailty on the Balance T program, participants were categorized in two frailty status levels; robustness (*n* = 14) and ‘at-risk’ of falls (*n* = 33).

**Table 2:** Demographics.

Characteristic	Number of Participants	Percentage
Total Enrolled	50	100 %
Gender		
Female	37	74 %
Male	13	26 %
Race		
White	44	88 %
African American	5	10 %
Asian	1	2 %
Ethnicity		
Non-Hispanic	50	100 %
Age		
51–60	1	2 %
61–70	3	6 %
71–80	25	50 %
81–90	21	42 %
Frailty Score		
0	14	29 %
1	22	44 %
2	9	18 %
3	5	10 %

### 3.1 Subjective improvement

After completing the 4-week Balance T home exercise regimen, participants’ subjective perceptions of balance improved compared to pre-Balance T exercise. Initially, 8 of 47 participants scored below 67 % on the ABC Scale, indicating a high likelihood of falling [10]. Following the exercise program, all but one of these eight participants showed a substantial improvement (32 %) in their ABC Scale scores, the mean (*n* = 8) post-exercise improvement was 24.1 ± 14.9 (range 10.6—45.6). The minimally detectable change (MDC) score for community-dwelling healthy older adults is unknown, but studies for individuals with Parkinson’s disease suggest an MDC of 13–30 % [10]. The change in ABC Scale scores of all 47 participants from pre (81.7 ± 17.2) to post (85.4 ± 12.7) was not statistically significant (*p* = < 0.11).

### 3.2 Physical improvement

**Fig. 2:**
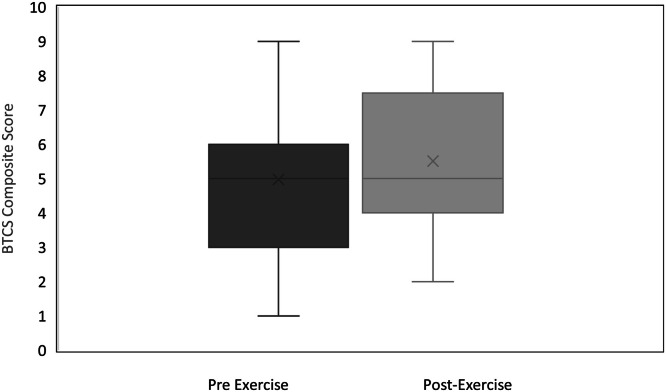
The Balance T Change Score (BTCS) is a novel battery of nine metrics capturing lower body strength, static and dynamic balance capacity. Scores range from 0 – 9 where 9 indicates greater balance. BTCS scores indicated significant improvement in Balance T Change Score (BTCS) scores from pre-exercise mean of 4.98 ± 2.05 to a post-exercise mean of 5.51 ± 2.15 (mean change = 0.53).

Balance performance, as measured by the SPPB, TUG, and CTSIB also improved significantly (Fig. 2). Normative data for community-dwelling older adults (CDOA) suggests normal SPPB scores range from 8.3 to 10.45, however, scores below 9 suggest an increased risk for developing a disability performing an activity of daily living (ADL) over the next 4 years [11]. Participants demonstrated significant improvement in SPPB scores, from a pre-exercise mean of 8.4 ± 1.7 to a post-exercise mean of 9.78 ± 1.6 (mean change = 1.38), which was both statistically significant (*p* = 1.45 × 10^–8^) and of substantial meaningful change (> 1.34) [10].

On the TUG test, scores greater than 13.5 s are considered abnormal for CDOA. Of the 11 participants with initially abnormal scores, seven showed normalization after the exercise program (mean change = 3.6 ± 1.2 s), three improved but remained abnormal (mean change = 2.5 ± 0.5 s), and one worsened (1.98 s slower). Overall, the TUG scores significantly decreased from a pre-assessment mean of 12.1 ± 1.9 to a post-exercise mean of 10.4 ± 2.7 (*p* = 2.8 × 10^–9^), representing a 14 % improvement. The MDC for CDOA is unknown but varies between 2.9 and 4.85 s across different populations such as persons suffering a stroke and those with Alzheimer’s or Parkinson’s Disease [8], [10], [12].

Scores on the CTSIB improved significantly from a pre-Balance T exercise regimen mean of 106 ± 15.7 to a post-Balance T exercise regimen mean of 110.6 ± 11.1 (*p* = 0.019). The MDC for the CTSIB is unknown.

### 3.3 Cohort analysis

We categorized the data in two subgroups indicating either ‘robustness’ (*n* = 14) or some level of frailty with a ‘risk of fall’ (*n* = 33). The at-risk participants experienced greater change in their ABC score than the robust participants upon completing the Balance T home exercise program for a mean change of 5.28 ± 12.9 (*p* = 0.001). All subjects improved their BTCS scores from Pre (4.98 ± 2) to (Post 5.5 ± 2.1) (*p* = 0.01) regardless of frailty rating. Although the mean scores for at-risk participants were always worse than that of robust participants for SPPB, TUG, and CTSIB the difference in improvement between the at-risk and robust were not statistically significantly different (*p* = ≥ 0.4).

### 3.4 Balance improvement as measured at home

**Fig. 3:**
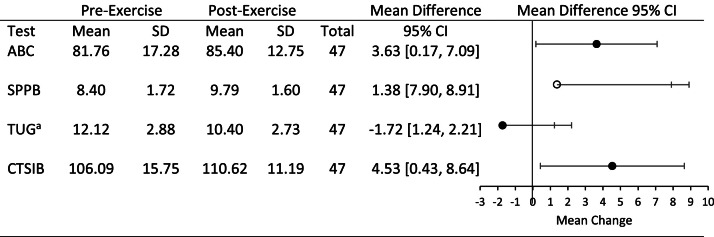
Mean and standard deviation scores in balance outcomes measured in the clinic before and after completing the 4-week Balance T home exercise program. ABC – Activity-specific Balance Confidence scale, higher scores indicate improvement; SPPB – Short Physical Performance Battery, higher scores indicate improvement; TUG – Timed Up and Go, lower score indicates improvement; CTSIB – Clinical Test of the Sensory Interaction on Balance, higher score indicates improvement. Open symbol denotes a clinically meaningful improvement.

Participants demonstrated significant improvement (*p* = 0.01) in Balance T Change Score from a pre-exercise mean of 4.98 ± 2.05 to a post-exercise mean of 5.51 ± 2.15 (mean change = 0.53, Fig. 3).

For the cohort analysis, the at-risk individuals significantly improved (*p* = 0.02) from a pre-assessment means of 4.9 ± 2.0 to a post-Balance T exercise mean of 5.5 ± 21. (mean change = 0.51). The robust participants also improved from a pre-exercise mean of 5.0 ± 2.1 to a post-exercise mean of 5.6 ± 2.1 (mean change = 0.6) though this was not statistically significant (*p* > 0.1). There was no difference in magnitude improvement of BTCS scores between the at-risk and robust subgroups (*p* > 0.4).

### 3.5 User opinion of home exercise with the balance T

**Fig. 4:**
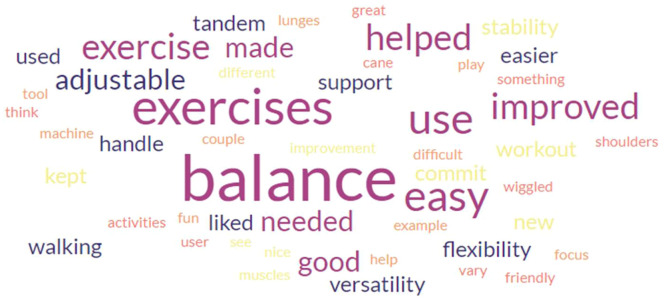
Word Cloud Capturing Positive Comments Written from Balance T User Opinion. Most users reported the Balance T improved their balance and helped build confidence in their ability to exercise.

In a post-exercise user opinion survey, participants indicated subjective improvement in their balance, pointing to their increased confidence and ability to perform specific movements that they were previously unable or unwilling to do pre-exercise. The user opinion survey showed 84 % of 45 participants thought the Balance T was either “very easy” or “easy” to use and 86 % of 44 responses indicated that users felt either “very safe” or “safe” when exercising with the Balance T. Additionally, 85 % of 40 users responded that the prescribed exercises were the correct amount of difficulty. When asked what users liked about the Balance T, the most frequently provided response was finding that the Balance T improved their balance and helped build confidence in their ability to exercise (Fig. 4). Users also reported that they liked the weekly phone calls and found it helpful to discuss the exercise program with a physical therapist (PT) during their participation. Finally, when users were asked what they did not like about the Balance T, the most frequently provided answer was “nothing”, followed by some mentions regarding adjusting the Balance T collar mechanism.

## 4 Discussion

The Balance T exercise program demonstrated significantly improved subjective balance and balance performance at clinically meaningful levels in most of the outcome measures collected. Not only did four weeks use of the Balance T improve balance, a key finding revealed the Balance T offered a greater magnitude improvement in those older adults with a worse rating of frailty. Those participants categorized as ‘at-risk’ for frailty demonstrated greater balance improvement. Additionally, a majority of the users (>80 %) reported the Balance T as easy and safe to use while exercising, and that the intensity was appropriately challenging. The high rate of compliance and positive feedback received from participants indicated that the Balance T and the accompanying exercise protocol were well-received and overall deemed beneficial to the participants.

Delivery of the Balance T exercise program was of a multifaceted design, utilizing in-person clinic visits, weekly phone calls, and self-administered balance testing. The combination of rehabilitation (home exercise program) coupled with weekly clinician phone likely was a significant contribution to ensure compliance and comprehension of the exercise program. The weekly phone calls allowed participants to discuss their weekly Balance T exercises including the impending change in the next set of exercises and any challenges or complications they experienced. This compliance we report is high, and a function of weekly contact from the PT. We believe this design offers great potential for increasing the accessibility of physical rehabilitation research at home and that our results are generalizable to the broader population of those in need for balance training. Our results further underscore the importance of targeted, accessible exercise interventions in maintaining mobility and improving balance in those at risk for fall among older adults. We are hopeful to investigate further efforts to deliver balance rehabilitation in the home as a fall risk management strategy.

In the context of aging research, distinguishing between varying degrees of frailty among older adults is critical for understanding variations in balance and mobility outcomes. Frailty, a clinical syndrome characterized by diminished strength, endurance, and physiologic function, places individuals at heightened risk for adverse health outcomes, including falls and disability [9]. Conversely, pre-frailty represents an intermediate state where individuals exhibit some but not all features of frailty, and are at an increased risk of progressing to frailty if not intervened upon. Additional research with a larger sample size to enable greater differentiating of these outcomes by frailty status may provide clearer insights into the effectiveness of the Balance T exercise device in improving balance across stages of frailty.

To our knowledge, the instruction of participants on how to self-administer and monitor improvement in balance was novel and has not been demonstrated to date. It appears the patient-collected data is valid given the evidence of meaningful change. Our results establish a ‘proof of principal’ that both completing a four-week home balance exercise program and measuring change can be safely done at home without the need for an in-person clinical visit. Considering the optimistic impressions captured from the User Opinion Survey, it may be valuable to correlate user opinion with patient-collected balance test scores.

Balance therapy and the intervention and measurement procedures in this study pose minimal risk to research participants are generally well-tolerated. The Balance T does not present any more risk than using a cane for walking and exercising with the Balance T is no riskier than performing home exercises as part of a clinician-prescribed rehabilitation program. Though none of the research participants reported any harms or adverse events, potential risks to participants include fatigue from completing the standing or walking balance exercises.

### 4.1 Limitation

Our choice to use an open-label, single-arm pre–post design was to efficiently evaluate the effect of the Balance T within-subjects, given the heterogeneity of balance impairment among individuals can be from multiple factors (i.e. neurological, age, musculoskeletal). This design minimizes between-subject variability and is well-suited for early-stage research. We acknowledge however, the absence of a control group limits our ability to exclude improvement in balance were instead related to time and not the exercises as open-label studies have limits causal inference. This will need to be addressed in future, controlled studies. We recognize the cohort analysis is unevenly distributed which limits our ability to confirm a significant difference in mean change (from pre- to post-exercise) between those categorized as “at-risk” versus “robust” frailty. A larger sample size would adequately address this. Additionally, we were unable to directly validate the patient-collected data at home. As video capture methods of gait kinematics mature, such technology will avail the necessary verification that patients who are at risk for fall can safely complete home balance exercises while also safely monitoring their progress. While our data support that four weeks of home balance exercises can lead to meaningful change, a duration of time supported by prior studies [13,14], we did not investigate how long these improvements might persist. Our creation of the BTCS, while novel, needs to be psychometric vetted as reliable and valid. Finally, although our data showed significant improvement in most balance outcomes, the amount of meaningful change of those outcome measures is unknown.

## 5 Conclusions

A four-week home exercise program using the Balance T device was feasible for both healthy and frail adults. Participants demonstrated improvements in balance performance, and frail individuals showed increased balance confidence. These preliminary findings support the potential of the Balance T device as a practical tool for home-based balance training.

More information about the Balance T can be found at https://balance-t.com/

## Declaration of generative AI and AI-assisted technologies in the writing process

During the preparation of this work the authors used Artificial intelligence (AI) in order to enhance clarity, tone, and structure of the writing. After using this tool, the authors reviewed and edited the content as needed and take full responsibility for the content of the published article.

## Précis

The Balance T device is an easy, safe and effective tool for improving both balance confidence and performance in older adults, with potentially greater benefits for those experiencing frailty.

## Funding

This work was supported by the Johns Hopkins AI and Technology Collaboratory for Aging Research, funded by the National Institute of Aging [P30AG073104]

## Proprietary interest statement

Drs Agrawal and Schubert developed the Balance T and share rights as co-owners of the patent (US20220241650A1) and co-owners of the Balance-T LLC*.*

## CRediT authorship contribution statement

**Courtney Walker:** Conceptualization, Formal analysis, Visualization, Writing – original draft, Writing – review & editing. **June Nicholas:** Investigation, Methodology, Writing – review & editing. **Crystal Szczesny:** Data curation, Formal analysis, Investigation, Methodology, Project administration, Writing – review & editing. **Jeremy Walston:** Conceptualization, Methodology, Writing – review & editing. **Yuri Agrawal:** Conceptualization, Funding acquisition, Methodology, Writing – review & editing. **Michael C Schubert:** Conceptualization, Formal analysis, Funding acquisition, Investigation, Methodology, Supervision, Validation, Writing – review & editing.

## Declaration of competing interest

The authors declare the following financial interests/personal relationships which may be considered as potential competing interests:

Michael C Schubert reports a relationship with Balance-T LLC that includes: equity or stocks. Yuri Agrawal reports a relationship with Balance-T LLC that includes: equity or stocks. Michael C Schubert has patent #US20220241650A1 issued to Balance-T LLC. Yuri Agrawal has patent #US20220241650A1 issued to Balance-T LLC. If there are other authors, they declare that they have no known competing financial interests or personal relationships that could have appeared to influence the work reported in this paper.

## Appendix Supplementary materials

Image, application 1

Image, application 2
