# Supplementary material for: “Balance T” device improves balance confidence and performance in repeated measures study
Source: J Frailty Aging. 2025 Nov 27;14(6):100113. doi: 10.1016/j.tjfa.2025.100113 (PMC12704060; doi:10.1016/j.tjfa.2025.100113)
Supplement: Supplementary file 1 [file mmc1.pdf]

Date: October 5, 2023
Principal Investigator: Ali Tehrani MD
Application No.: IRB00364181

Balance-T Exercises

IMPROVE YOUR BALANCE AND AGILITY

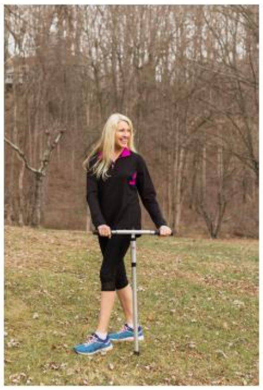

These exercises are designed to improve balance whether you are young, old, injured or healthy

User Opinion Survey

1. How easy would you say the Balance T was to use? Choose one:
   a. Very Easy   b. Easy   c. Neutral   d. Hard   e. Very Hard

2. How safe did you feel when exercising with the Balance T? Choose one:

3. a. Very Easy   b. Easy   c. Neutral   d. Hard   e. Very Hard

4. What type of users would you recommend using the Balance T? Circle all that apply:

Older Adults?   Young or middle-aged adults?   Those training in sports?

5. How much would you be willing to pay for the Balance T? ________________________

6. How likely are you to recommend the Balance T to your friends or family? Choose one:
   a. 0%   b. 25%   c. 50%   d. 75%   e. 100%

7. What did you like most about the Balance T?

8. What did you not like about Balance T or what would you change about Balance T?

9. Did you find that the exercises done with Balance T were…..
   a. too easy?   c. too hard?   c. the correct amount of challenge?

10. Did your balance improve as a result of participating in the program? If so, in what ways did it improve?

11. How likely are you to continue to use the Balance T for your own benefit?

12. Please share any other comments that you have about the Balance T with respect to its value.
