# Supplementary material for: “Balance T” device improves balance confidence and performance in repeated measures study
Source: J Frailty Aging. 2025 Nov 27;14(6):100113. doi: 10.1016/j.tjfa.2025.100113 (PMC12704060; doi:10.1016/j.tjfa.2025.100113)
Supplement: Supplementary file 2 [file mmc2.pdf]

Balance T Device Study Exercise Program

WEEK ONE

Exercise 1 • Seated, hold the Balance T bar in front of you with post on the ground • Rotate the handles toward and away from you, alternating right then left • Continue for 2 minutes

Exercise 2 • Seated, hold the Balance T bar in front of you with post on the ground • Push both hands forward and backward • Continue for 2 minutes.

Exercise 3 • Scoot to the edge of your seat • Push the entire Balance T forward as you lean forward and stand up • Continue for 2 minutes

Exercise 4 • Stand with feet shoulder-width apart • Hold the Balance T, rotate the handles toward and away from you • Alternate right then left • Continue for 2 minutes

Exercise 5 • Stand with feet shoulder-width apart • Hold the Balance T, alternate tilting the handle up and down • Continue for 2 minutes

Exercise 7 • Stand with feet shoulder-width apart • Hold the Balance T and shift your weight to the left foot as your head tilts to the right. Continue for one minute. Now shift your weight to the right as you head tilts to the left. Continue for one minute.

> 1st PROGRESSION • Rock side to side from right foot to left foot, no pause. Continue for 2 minutes.
